# Statistical Frailty Modeling for Quantitative Analysis of Exocytotic Events Recorded by Live Cell Imaging: Rapid Release of Insulin-Containing Granules Is Impaired in Human Diabetic *β*-cells

**DOI:** 10.1371/journal.pone.0167282

**Published:** 2016-12-01

**Authors:** Giuliana Cortese, Nikhil R. Gandasi, Sebastian Barg, Morten Gram Pedersen

**Affiliations:** 1 Department of Statistical Sciences, University of Padua, Padua, Italy; 2 Department of Medical Cell Biology, Uppsala University, Uppsala, Sweden; 3 Department of Information Engineering, University of Padua, Padua, Italy; University of Bremen, GERMANY

## Abstract

Hormones and neurotransmitters are released when secretory granules or synaptic vesicles fuse with the cell membrane, a process denoted exocytosis. Modern imaging techniques, in particular total internal reflection fluorescence (TIRF) microscopy, allow the investigator to monitor secretory granules at the plasma membrane before and when they undergo exocytosis. However, rigorous statistical approaches for temporal analysis of such exocytosis data are still lacking. We propose here that statistical methods from time-to-event (also known as survival) analysis are well suited for the problem. These methods are typically used in clinical settings when individuals are followed over time to the occurrence of an event such as death, remission or conception. We model the rate of exocytosis in response to pulses of stimuli in insulin-secreting pancreatic *β*-cell from healthy and diabetic human donors using piecewise-constant hazard modeling. To study heterogeneity in the granule population we exploit frailty modeling, which describe unobserved differences in the propensity to exocytosis. In particular, we insert a discrete frailty in our statistical model to account for the higher rate of exocytosis in an immediately releasable pool (IRP) of insulin-containing granules. Estimates of parameters are obtained from maximum-likelihood methods. Since granules within the same cell are correlated, i.e., the data are clustered, a modified likelihood function is used for log-likelihood ratio tests in order to perform valid inference. Our approach allows us for example to estimate the size of the IRP in the cells, and we find that the IRP is deficient in diabetic cells. This novel application of time-to-event analysis and frailty modeling should be useful also for the study of other well-defined temporal events at the cellular level.

## Introduction

Novel methods for the study of cell biological processes produce unprecedented data to be analyzed. To maximize the information that can be extracted from the experimental results, appropriate and advanced statistical analytical methods should be exploited. Recent microscopy techniques, in particular total internal reflection fluorescence (TIRF) microscopy, have made it possible to visualize single exocytotic events in neurons and endocrine cells [1–8]. Exocytosis is the process during which the lipid membranes of neurotransmitter-filled synaptic vesicles (in neurons), or hormone-containing secretory granules (in endocrine cells), fuse with the cell membrane, which allows the signalling molecules contained within the granule to escape to the extracellular space [9].

Such imaging data has given deep insight into the molecular and dynamical regulation of exocytosis. However, to our knowledge, these single-granule data have until now been analyzed by counting the (cumulative) number of events over all observed granules and cells, sometimes followed by simple curve fitting [3, 4, 10]. Thus, more rigorous methods for quantification and analysis of imaging data of exocytosis are needed [6]. We propose here that the detailed temporal information contained in this type of data allows for statistical analysis using tools from time-to-event (also known as survival or failure time) analysis. These methods are typically used for clinical or demographic data where individuals are followed until a certain event of interest, such as death, onset of disease, conception, first-time marijuana use, etc. [11–13]. Another common area of their application is reliability engineering where the interest is the time to failure of an instrument or machine. Since the structure in such data is similar to the live cell imaging exocytosis data of interest here, it allows us to apply these well-established statistical methods on completely different biological and temporal scales.

Insulin is released from pancreatic *β*-cells in response to various stimuli, with glucose being the physiologically most important. Disturbed insulin secretion is now recognized as a central player in the development of diabetes, a devastating disease which is reaching epidemic proportions [14, 15]. Glucose is transported into the *β*-cells where it triggers a complex cascade of events leading to cell depolarization and electrical activity. As a result, voltage-dependent Ca^2+^ channels open, promoting Ca^2+^ influx, and the increase in intracellular Ca^2+^ levels cause exocytosis of insulin-containing secretory granules [16]. Insulin secretion is biphasic in response to a sustained glucose stimulus; a large peak of insulin release is followed by a second phase where insulin is released in distinct pulses [17]. Importantly, biphasic insulin release is disturbed in diabetes [18], which has been suggested to have its origin within the pancreatic *β*-cells [19], likely because of dysfunctional exocytosis [20–22].

It was early proposed that heterogeneous release propensities of the insulin-containing granules could underlie the biphasic secretion pattern. In this hypothesis a small pool of granules is released to yield the first peak of insulin whereas slower release of other granules produce the second phase of secretion [23]. More recent results in various endocrine cells [24–26] showed that a sustained elevation of intracellular Ca^2+^ levels could produce a phasic exocytosis pattern as measured by membrane capacitance recordings reflecting whole-cell release. Also, repeated or sustained depolarizations, which promote Ca^2+^ entry via voltage-dependent Ca^2+^ channels, triggered phasic capacitance patterns, even when investigated as a function of Ca^2+^ entry [16, 27, 28]. These patterns were interpreted as the results of depletion of a small immediately releasable pool (IRP) of granules followed by slower release from a larger pool. Various mathematical models of granule pools and exocytosis were developed based on these and similar results, with the scope of reproducing and simulating typical behavior, in order to investigate the underlying biological mechanisms [25, 29–33]. However, the aim of such mathematical models is not to extract information from raw experimental data. For such a task, statistical methods are needed.

We propose and show here that survival analysis methods can be advantageously applied to cell biological data to provide statistically sound results on completely different biological and temporal scales than their typical areas of application. In particular, we apply time-to-event analysis to exocytosis data from healthy and diabetic human *β*-cells to quantify hazards (rates of exocytosis) and heterogeneity. In survival analysis, univariate frailty modeling is a method to take into account unobserved differences in hazards between individuals [13, 34]. In the present context, imaging of the secretory granules can not reveal their release propensity, i.e., whether they belong to the IRP. Based on the biological findings and interpretations cited above, we thus allow for heterogeneity by including frailties in our statistical model. This approach allows us to estimate the size of the IRP directly from single-granule exocytosis data. We estimate that the IRP is smaller in diabetic cells, and that exocytosis is less tightly controlled by depolarizing K^+^ pulses compared to healthy cells.

## Materials and Methods

### Data description

Human pancreatic islets were provided by the Nordic Network for Clinical Islet Transplantation (Uppsala, Sweden) with full ethical approval (Regionala Etikprövningsnämden, Uppsala). Islets were dissociated into single cells in 0.0025% trypsin in Ca^2+^/Mg^2+^-free cell dissociation buffer (ThermoFisher) for 3-5 minutes and seeded onto polylysine-coated glass coverslips, and cultured in CMRL 1066 medium containing 5.6 mM glucose, 10% fetal calf serum (FCS), and 2 mM L-glutamine, streptomycin (100 *μ*g/ml), penicillin (100 *μ*g/ml). Seeded cells were infected using adenovirus encoding the granule marker NPY-mCherry (Neuropeptide Y fused to the red fluorescent protein mCherry; [5, 35]) and imaged 24-36 hours later. Insulin-containing secretory granules in pancreatic *β*-cells from 3 healthy (11 cells) and 2 diabetic (8 cells) donors were imaged using total internal reflection fluorescence (TIRF) microscopy at a frame rate of 10 Hz, with excitation at 561 nm and emission at 590-630 nm. Cells were bathed in (in mM) 138 NaCl, 5.6 KCl, 1.2 MgCl2, 2.6 CaCl2, 10 D-glucose and 5 HEPES (pH 7.4 with NaOH), 2 *μ*M forskolin and 200 *μ*M diazoxide. The latter prevents glucose-dependent depolarization by opening ATP-dependent K^+^-channels. Forskolin, which increases intracellular cyclic-AMP, was routinely included to increase the number of primed granules available for exocytosis.

**Fig 1:**
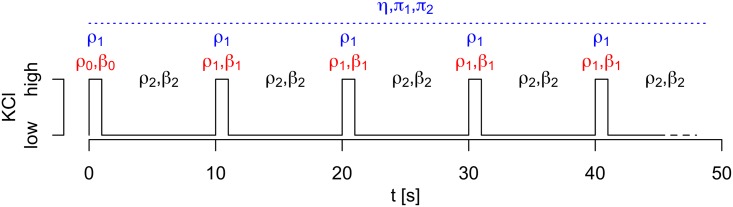
Stimulation protocol and related model parameters. An indication of the high-concentration K^+^ pulses (1 s) interspersed by 9 seconds of rest. The parameters common to the two statistical models are indicated in black (for the pulses following the first pulse) and gray (for the rest intervals). The two models have different parameters for the first pulse. In the Poisson model (red), the baseline rate and effect of diabetes is allowed to be different during the first pulse compared to subseequent pulses (black). In the frailty model (blue), the baseline parameters are the same during all the pulses, but additional parameters (*η*, *π*_1_, *π*_2_) describing the frailty distribution are included. These additional parameters are not restricted to a certain time interval. See main text for detailed descriptions of the statistical models.

Exocytosis was then evoked by ten 1-second long pulses of local application of high concentrations of K^+^ (75 mM KCl equimolarly replacing NaCl), interspersed by 9-seconds long rest intervals (Fig 1). The K^+^ pulses depolarize the cellular membrane potential within ∼50 ms (unpublished observation), which opens voltage-dependent Ca^2+^ channels and the resulting Ca^2+^ influx triggers exocytosis. The rate of exocytosis is therefore expected to be higher during, compared to between, K^+^ pulses. All experiments were carried out with constant buffer perifusion at 32°C. Exocytosis events were found manually as sudden disappearance of labeled granules.

We considered the granules within a cell as a cluster of statistical units indexed by *j* = 1, …, *J*. Our data contains *J* = 19 clusters corresponding to the 19 cells, i.e., the terminology ‘cluster’ refers to a structure in the data: the observations obtained from the granules (the statistical units) in a cell. Cluster *j* had *n*_*j*_ observations, representing the granules in the cell, with index *i* = 1, …, *n*_*j*_. For granule *i* in cell *j* we observed either the time of exocytosis, ${\tilde{t}}_{ij}$, or the *censoring time* *c*_*ij*_, i.e., the last observed time. In these data, *c*_*ij*_ is the time when the experiment ended, and is thus the same for all granules (so-called administrative censoring). Censoring precludes the observation of exocytosis that might have occurred at a later time. Thus, the observed data are the pairs (*t*_*ij*_, *d*_*ij*_), where *t*_*ij*_ are the realizations of the observed survival time $T_{ij}=\min\left({\tilde{T}}_{ij},C_{ij}\right)$, and *d*_*ij*_ is the observed indicator from $D_{ij}=I\left({\tilde{T}}_{ij}<C_{ij}\right)$ that tells whether a granule underwent exocytosis (*d*_*ij*_ = 1) or was censored (*d*_*ij*_ = 0). This form of the data is typical for time-to-event data.

### Poisson regression modelling

For the analysis of the exocytosis data, we proceeded progressively. Poisson regression neglecting heterogeneity was exploited to investigate whether the data can be described with a time-varying, piecewise constant hazard, although biologically unlikely as discussed below. This approach also serves as the basis for the formulation of the frailty model in the next subsection, as well as a reference frame for the results that follow.

We assumed that the rate (or *hazard function*) of exocytosis *μ*(*t*) is piecewise constant. The hazard was assumed to be constant during each pulse and during each interval between two consecutive pulses, but it may vary from one pulse to another, and from pulses to intervals between pulses. Model selection led to three parameters, *ρ*_0_, *ρ*_1_, *ρ*_2_, estimating, respectively, the rate during the first pulse, the rate during the other pulses, and the rate between pulses. The model also included a covariate *X* indicating whether the cell came from a healthy (*X* = 0) or diabetic donor (*X* = 1). The effect of diabetes was assumed to be time-varying in a piecewise-constant fashion corresponding to the hazard, i.e., we considered three parameters *β*_0_, *β*_1_, *β*_2_ describing the effects, respectively, during the first pulse, during the following pulses, and between pulses. The hazard function was thus modeled as

$$\mu \left(t|X\right)=\left\{\begin{array}{cc} \rho_{0}e^{\beta_{0}X}=e^{\alpha_{0}+\beta_{0}X}, & 0\leq t<1\;\text{seconds},\\ \rho_{1}e^{\beta_{1}X}=e^{\alpha_{1}+\beta_{1}X}, & s\leq t<s+1\;\text{for}\;\text{some}\;s=10,20,30,\ldots \;\text{seconds},\\ \rho_{2}e^{\beta_{2}X}=e^{\alpha_{2}+\beta_{2}X}, & s+1\leq t<s+10\;\text{for}\;\text{some}\;s=0,10,20,\ldots \;\text{seconds}, \end{array}\right.$$

where *α*_*k*_ = log *ρ*_*k*_, and *k* = 0, 1, 2 indicate whether *t* falls in the first pulse (*k* = 0), in one of the following pulses (*k* = 1), or between pulses (*k* = 2) (Fig 1). In particular, we were interested in the question of whether the rate of exocytosis was different between healthy and diabetic cells, and if this difference was restricted to the first pulse.

Since only a small fraction of granules exhibited exocytosis during the experiments, Poisson modeling can be used to describe the data [36]. We used the R [37] function glm to perform the analysis. To get cluster-corrected standard errors and Wald-type confidence intervals (which are calculated from standard errors) for the parameter estimates, we used the robust sandwich estimator (see Eq 5 below) based on R code by Arai [38]. Cox proportional hazards modeling can also investigate the time-dependent effect of diabetes by including time-varying parameters [12], but the baseline hazard function is estimated nonparametrically. When we applied this model, it gave virtually identical results to the Poisson model for the diabetes effect.

### Frailty modelling of two pools of granules

The interpretation of the selected Poisson model is that for any granule the rate of exocytosis is higher during the first pulse than during the following pulses, for example because of a reduction in the triggering Ca^2+^ signal as a result of Ca^2+^ channel inactivation. Such an interpretation is biologically unlikely, since the 9 sec interval between pulses is sufficiently long to allow reactivation of Ca^2+^ currents [39]. Thus, if anything, the Ca^2+^ levels should build up from one K^+^ pulse to the next, which would increase the rate of exocytosis for pulses later in the train.

An alternative and widely used explanation is to attribute the greater amount of release in the beginning of the stimulus protocol to an immediately releasable pool (IRP) of granules that have a much higher intrinsic rate of exocytosis than the remaining, non-IRP, granules [21, 23]. Once this pool is empty, exocytosis proceeds at a slower pace.

Imaging of the labeled granules can not reveal whether a given granule belongs to the IRP, nor can the size of the IRP be seen from the microscopy images. Statistically, we can handle this scenario by introducing a (non-observable) Bernoulli variable *Y*, where the realization *Y*_*ij*_ is equal to 1 when granule *i* of cell *j* belongs to the IRP and 0 otherwise. To allow for different sizes of the IRP in healthy and diabetic cells we assume that the probability *P*(*Y* = 1|*X*) = *π*_*X*_ depends on the diabetes-covariate *X*.

Exocytosis of an IRP granule is assumed to occur with a rate that is *η* times higher than the baseline rate describing non-IRP exocytosis. This assumption is described by a discrete frailty *Z*, which takes the value *η* when *Y* = 1, and *Z* = 1 otherwise. The resulting frailty model is thus

$$\mu \left(t|X,Z\right)=Z\mu_{0}\left(t|X\right),\;P\left(Z=\eta |X\right)=\pi_{X},\;P\left(Z=1|X\right)=1-\pi_{X}.$$ (1)

The baseline hazard *μ*_0_ is piecewise constant with rate *ρ*_1_*e*^*β*_1_*X*^ = *e*^*α*_1_+*β*_1_*X*^ during K^+^ pulses and rate *ρ*_2_*e*^*β*_2_*X*^ = *e*^*α*_2_+*β*_2_*X*^ between pulses. Thus, *β*_1_ and *β*_2_ describe effects of diabetes on the rates-of-exocytosis during and between K^+^ pulses, respectively. Note that in contrast to the Poisson model, the baseline rate is assumed to be identical during the first and the subsequent K^+^ pulses (Fig 1).

In time-to-event analysis, one of the main overall summary measures of interest is the survival probability *S*(*t*) = *P*(*T* ≥ *t*), or, equivalently, the cumulative incidence probability defined as *F*(*t*) = *P*(*T* < *t*) = 1 − *S*(*t*). *S* can be estimated in a model-free, nonparametric way using for example the Kaplan-Meier estimator [11]. For the frailty model (1), the marginal survival function is given as

$$S\left(t|X\right)=\pi_{X}e^{-\eta M_{0}\left(t|X\right)}+\left(1-\pi_{X}\right)e^{-M_{0}\left(t|X\right)},$$

where $M_{0}\left(t|X\right)=\int_{0}^{t}\mu_{0}\left(t|X\right)$ is the cumulative baseline hazard [13]. This expression is a mixture of the survival functions of an IRP granule and a non-IRP granule, weighted by their respective probability to be observed.

We construct the likelihood function under the *working independence assumption* [40, 41]. This means that for the time being we ignore the clustered structure of the data caused by the correlation between granules within the same cell. Following the work of Yu & Peng [41] on cure models, a particular type of discrete frailty model with *η* = 0, we then integrate the frailty out to obtain a marginal likelihood function. The resulting marginal *independence* log-likelihood, *l*_*I*_, gives valid maximum likelihood estimate (MLE) ${\hat{\theta }}_{I}$ of the parameter vector *θ*, but the inverse of the observed Hessian of the independence log-likelihood, ${\hat{H}}^{-1}$, does not yield valid estimates of e.g. standard errors [40, 42]. Thus, in order to construct confidence intervals or perform inferential tests, corrections must be introduced.

Under the independence assumption, the log-likelihood, conditional on the frailty *Z*, is given as the sum of the individual contributions,

$${\tilde{\ell }}_{I}\left(\theta |t_{ij},d_{ij},X_{ij},Z_{ij}\right)=\sum_{j=1}^{J}\sum_{i=1}^{n_{j}}{\tilde{\ell }}_{ij}\left(\theta |t_{ij},d_{ij},X_{ij},Z_{ij}\right),$$ (2)

with

$${\tilde{\ell }}_{ij}=\log\left[\mu {\left(t_{ij}|X_{ij},Z_{ij}\right)}^{d_{ij}}e^{-M(t_{ij}|X_{ij},Z_{ij})}\right],$$

where *M* is the cumulative hazard function,

$$M\left(t|X,Z\right)=\int_{0}^{t}\mu \left(s|X,Z\right)ds=Z\int_{0}^{t}\mu_{0}\left(s|X\right)ds=ZM_{0}\left(t|X\right),$$

and *M*_0_ the cumulative baseline hazard. Since *Z* is unobservable, it must be integrated out of the log-likelihood Eq (2) to obtain the MLE. This procedure yields the marginal (unconditional on *Z*) independence log-likelihood

$$\ell_{I}\left(\theta |t_{ij},d_{ij},X_{ij}\right)=\sum_{j=1}^{J}\sum_{i=1}^{n_{j}}\ell_{ij}\left(\theta |t_{ij},d_{ij},X_{ij}\right),$$ (3)

where

$$\begin{array}{c} \ell_{ij}(\theta |t_{ij},d_{ij},X_{ij})=\\ \;\text{log}\left[\mu_{0}{\left(t_{ij}|X_{ij}\right)}^{d_{ij}}\left(\pi_{X_{ij}}\eta^{d_{ij}}e^{-\eta M_{0}(t_{ij}|X_{ij})}+\left(1-\pi_{X_{ij}}\right)e^{-M_{0}(t_{ij}|X_{ij})}\right)\right] \end{array}$$ (4)

is found by averaging the likelihood function with respect to *Z* [13]. Given the data, *ℓ*_*I*_ can then be maximized to yield the MLE ${\hat{\theta }}_{I}$.

A commonly used approach to correct for clustering is to estimate the variance-covariance matrix using the so-called *robust* or *sandwich* estimator [40]

$$R={\hat{H}}^{-1}\hat{V}{\hat{H}}^{-1},\;\hat{V}=\sum_{j}^{}U_{j}\left({\hat{\theta }}_{I}\right)U_{j}{\left({\hat{\theta }}_{I}\right)}^{\prime },$$ (5)

where

$$U_{j}\left(\theta \right)=\sum_{i}U_{ij}\left(\theta \right)=\sum_{i}\frac{\partial }{\partial \theta }\ell_{ij}\left(\theta |t_{ij},d_{ij},X_{ij}\right)$$

is the score contribution from cluster *j*. From R, robust standard errors for ${\hat{\theta }}_{I}$, $SE_{{\hat{\theta }}_{I}}$, and correct Wald-type 95% confidence intervals, ${\hat{\theta }}_{I}\pm 1.96\;SE_{{\hat{\theta }}_{I}}$, can be obtained. However, Wald tests are not reliable for testing null hypothesis with parameters on the boundary of the parameter space (e.g., *π*_*X*_ = 0). Further, Wald-type inference can be difficult to interpret when covariates are highly correlated, and inference based on the likelihood ratio is preferable in finite samples [40].

In order to calculate valid likelihood-based confidence intervals, and perform likelihood ratio tests, Chandler & Bate [40] proposed to adjust the independence likelihood in order to obtain an *adjusted* log-likelihood function *ℓ*_*A*_ that has the same MLE as *ℓ*_*I*_, but has the ‘correct’ observed Hessian ${\hat{H}}_{A}$, i.e., the sandwich estimator in Eq (5) is obtained as the inverse of the observed Hessian, ${\hat{H}}_{A}=-R^{-1}$. This can be obtained by defining [40]

$$\ell_{A}\left(\theta \right)=\ell_{I}\left(C\left(\theta -{\hat{\theta }}_{I}\right)+{\hat{\theta }}_{I}\right),$$ (6)

where ${\hat{\theta }}_{I}$ maximizes *ℓ*_*I*_, and *C* = *N*^−1^ *N*_*A*_ with $N^{\prime }N=\hat{H}$ and $N_{A}^{\prime }N_{A}={\hat{H}}_{A}=-R^{-1}$. The matrix square-roots *N* and *N*_*A*_ are conveniently constructed from the spectral decompositions of $\hat{H}$ and $-R^{-1}$ [40]. Likelihood ratio tests, e.g. of the null hypothesis that a given parameter is equal to zero, say *θ*_*k*_ = 0, can then be performed by comparing quantiles of the $\chi_{1}^{2}$ distribution to the log likelihood ratio statistics $\Lambda_{A}=2\left(\ell_{A}\left({\hat{\theta }}_{I}\right)-\ell_{A}\left(\tilde{\theta }\right)\right)$, where $\tilde{\theta }$ maximizes *ℓ*_*A*_ under the constraint *θ*_*k*_ = 0. When performing tests against a null hypothesis with parameters on the boundary of the parameter space, e.g., testing *π*_0_ = 0 or *π*_1_ = 0, Λ_*A*_ is compared to the quantiles of the mixture distribution $(\chi_{0}^{2}+\chi_{1}^{2})/2$ [43]. This amounts to performing a one-sided test.

As recommended by Chandler & Bate [40], we chose a reparameterization that led to symmetric log-likelihoods, as verified by the symmetry of the estimated confidence intervals. In particular, we estimated the parameter vector $\theta =\left(\alpha_{1},\alpha_{2},\beta_{2},\tilde{\eta },{\tilde{\pi }}_{0},{\tilde{\pi }}_{1}\right)=\left(\log\rho_{1},\log\rho_{2},\beta_{2},\log\eta ,\sqrt{\pi_{0}},\sqrt{\pi_{1}}\right)$, and calculated confidence intervals and performed log likelihood ratio tests for *θ* based on *ℓ*_*A*_. For ease of interpretations, inferential results are presented for the original parameters using the inverse transformations of point and interval estimates. Calculations were performed in R [37]. Optimization of *ℓ*_*I*_ was done using the nlminb function. Numerical approximations to $\hat{H}$ and ${\hat{U}}_{j}$ were found using the hessian and grad functions from the numDeriv R package [44]. Spectral decompositions were obtained using the eigen R function.

## Results

### Time-varying, piecewise-constant hazard Poisson analysis

As explained in the Methods, we assume a piecewise constant hazard

$$\mu \left(t|X\right)=\left\{\begin{array}{cc} \rho_{0}e^{\beta_{0}X}=e^{\alpha_{0}+\beta_{0}X}, & 0\leq t<1\;\text{seconds},\\ \rho_{1}e^{\beta_{1}X}=e^{\alpha_{1}+\beta_{1}X}, & s\leq t<s+1\;\text{for}\;\text{some}\;s=10,20,30,\ldots \;\text{seconds},\\ \rho_{2}e^{\beta_{2}X}=e^{\alpha_{2}+\beta_{2}X}, & s+1\leq t<s+10\;\text{for}\;\text{some}\;s=0,10,20,\ldots \;\text{seconds}, \end{array}\right.$$

where *ρ*_*k*_ and *β*_*k*_ model baseline hazards and effects of diabetes, respectively, and the subscripts *k* = 0, 1, 2 refer to, respectively, the first pulse, the following pulses and the intervals between pulses. In the Poisson formulation, the parameters *α*_*k*_ = log(*ρ*_*k*_) and *β*_*k*_, *k* = 0, 1, 2 are estimated. However, to facilitate the interpretation of the baseline rates, inferential results are reported for *ρ*_*k*_ and *β*_*k*_, *k* = 0, 1, 2 (Table 1).

**Table 1:** Estimated parameters using Poisson modeling.

Parameter	estimate	95% CI	*p*-value	*p*-value (no clustering)
*ρ*_0_	0.0176	(0.0040, 0.0781)	<10^−11^	<10^−11^
*ρ*_1_	0.0013	(0.0004, 0.0040)		
*ρ*_2_	0.0003	(0.0001, 0.0005)	0.006	<10^−5^
*β*_0_	-1.48	(-3.54, 0.57)	0.157	0.046
*β*_1_	-0.09	(-1.81 1.62)	0.914	0.858
*β*_2_	0.98	(-0.04, 2.01)	0.060	0.0006

Wald-type 95% confidence intervals (CI) and *p*-values are based on the sandwich estimator R and *t*-tests. For *β*_*k*_, the *p*-values refer to the null-hypotheses *β*_*k*_ = 0. For *ρ*_0_ and *ρ*_2_, the *p*-values refer to the null-hypotheses *ρ*_0_ = *ρ*_1_ and *ρ*_2_ = *ρ*_1_, respectively. The last column shows naive *p*-values ignoring clustering.

**Fig 2:**
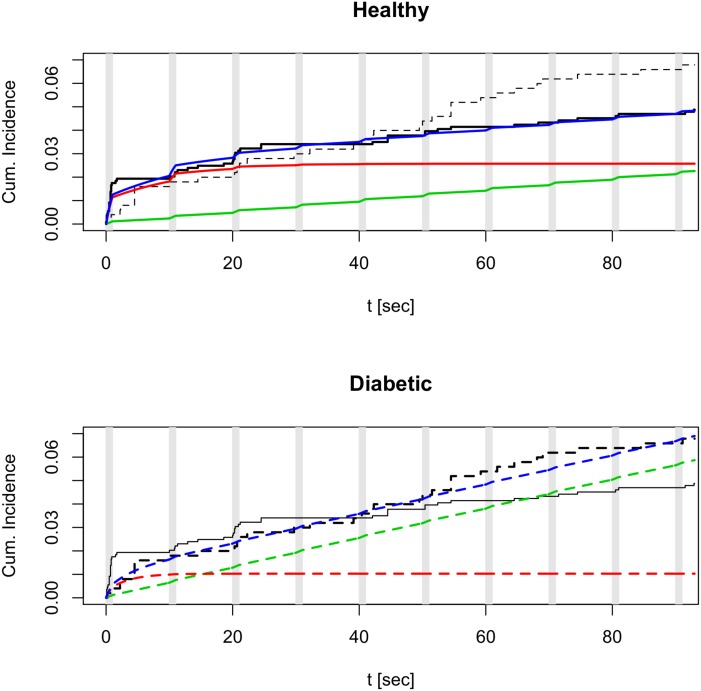
Estimated cumulative incidence probabilities. The curves represent the estimated probabilities of an exocytotic event before time *t* (the cumulative incidence) for a given granule in healthy (upper panel, full curves) or diabetic (lower panel, dashed curves) *β*-cells. The black curves are obtained from model-free, non-parametric Kaplan-Meier estimates, which, for comparison, are shown in both panels. Steps in these curves correspond to exocytotic events. For the frailty model we show the marginal estimate (blue), and the estimates conditional on the frailty, *Z* = *η* (IRP granules; red; scaled by *π*_*X*_) or *Z* = 1 (non-IRP granules; green; scaled by 1 − *π*_*X*_). The gray vertical lines indicate the K^+^ pulses.

The estimated rate related to the first pulse (${\hat{\rho }}_{0}=0.176$ s^−1^) was found to be significantly greater (about 14-fold) than the estimate related to the other pulses (${\hat{\rho }}_{1}=0.0013$ s^−1^; *p* < 10^−11^). As expected, the estimated rate between pulses (${\hat{\rho }}_{2}=0.0003$ s^−1^) was significantly lower (about 6-fold) than the rate during stimuli, reflecting that exocytosis mainly occur when Ca^2+^ channels open in response to the depolarizing K^+^ pulses. Interestingly, diabetes had no statistically significant effect on the hazard during pulses, though there was a tendency towards a reduced rate (∼75% reduction) of exocytosis during the first pulse in diabetic cells (*p* = 0.157, ${\hat{\beta }}_{0}=-1.48$, $\exp({\hat{\beta }}_{0})=0.23$). This reduced rate was however poorly estimated as reflected by the large confidence interval. On the contrary, between pulses the rate of exocytosis was 2-3 fold *higher* in diabetic cells than in healthy cells (*p* = 0.060, ${\hat{\beta }}_{2}=0.98$, $\exp({\hat{\beta }}_{2})=2.66$). We note that all the tests, except for *β*_1_, (erroneously) show significance if clustering is ignored. In summary, whereas healthy cells showed a prominent peak of exocytosis in response to the first pulse followed by bursts of release synchronized with the stimulating pulses, exocytosis occurred less well controlled by the stimuli in diabetic cells, as seen from nonparametric estimates of the cumulative incidence functions (Fig 2, black curves). These results correspond well to clinical characteristics of diabetes, where biphasic insulin secretion is disturbed [18].

### Frailty modeling of granule heterogeneity

The model presented in the previous subsection assume that all granules follow the same hazard function describing the rate of exocytosis. The obtained results suggest that this hazard declines from the first to subsequent stimulus pulses, and thus, that the peak of secretion is caused by a decrease in the rate of exocytosis. Alternatively, the peak of secretion is often attributed to a distinct *immediately releasable pool* (IRP) of granules that undergo exocytosis more rapidly that the non-IRP granules.

To account for a heterogeneous granule population, we introduced a discrete frailty variable *Z* that modeled the probability (*π*_*X*_) of a granule to belong to the IRP and the fold-increase in exocytosis rate in the IRP via the parameter *η*. The size of the IRP is hence described by *π*_*X*_, which was allowed to vary between healthy and diabetic cells. As described in the Methods, *Z* is a non-observable variable since we cannot *a priori* identify the granules that belong to the IRP.

In order to estimate the parameters in the model, we integrate *Z* out to obtain the marginal independence likelihood function *ℓ*_*I*_, which is then maximized. The resulting maximum likelihood estimates of the parameters are reported in Table 2. The Hessian of the independence likelihood function *ℓ*_*I*_ does not provide valid estimates for standard errors since it neglects the clustered structure of the data caused by the correlation between granules within the same cell. In order to perform valid inference, we adjust *ℓ*_*I*_ to obtain the adjusted likelihood function *ℓ*_*A*_, which permits us to construct confidence intervals from the log-likelihood statistics, and to perform ordinary log-likelihood ratio tests taking clustering into consideration [40] (Table 2).

**Table 2:** Estimated parameters using frailty modeling.

Parameter	MLE	95% CI	*p*-value	*p*-value (no clustering)
*ρ*_1_	0.00117	(0.00079, 0.00174)		
*ρ*_2_	0.00014	(0.00011, 0.00019)	0.0005	4 ⋅ 10^−7^
*β*_2_	1.43	(0.98, 1.84)	0.038	0.0002
*η*	499.5	(321.7, 773.4)	<0.0001	<10^−7^
*π*_0_	0.026	(0.013, 0.044)	0.022	<10^−7^
*π*_1_	0.010	(0.00003, 0.035)	0.052	0.008

Maximum likelihood estimates (MLE) are based on the independence likelihood function *ℓ*_*I*_. The 95% confidence intervals (CI) and tests of hypotheses are based on the log likelihood ratio statistic Λ_*A*_ obtained from the adjusted log-likelihood function *ℓ*_*A*_. For *ρ*_2_, the *p*-value refers to the null hypothesis *ρ*_1_ = *ρ*_2_. The last column shows *p*-values based on log likelihood ratio test using *ℓ*_*I*_ ignoring clustering.

In contrast to the Poisson model, we assumed that the rate of exocytosis, for a given granule (conditional on the frailty *Z*), was different during, compared to between, K^+^ pulses, but that the hazard was independent of the pulse number, i.e., *ρ*_0_ = *ρ*_1_ (Fig 1). Based on the Poisson model, and after performing model selection in the frailty formulation, we assumed that diabetes did not influence the rate of exocytosis during pulses, i.e., *β*_1_ = 0. However, diabetes was allowed to have an effect on the rate of exocytosis between pulses. As shown in Fig 2, the model provides a good overall fit to the data.

Our results (Table 2) concerning the estimated frailty parameter ${\hat{\pi }}_{0}$ suggest that the size of the IRP in healthy *β*-cells is significantly greater that zero (*p* = 0.022) and amounts to ∼2.6% of the docked granules. Note that if clustering is (erroneously) neglected, the significance of the test becomes extremely high (*p* < 10^−7^). In contrast, in diabetic *β*-cells, the estimated IRP size, as measured by ${\hat{\pi }}_{1}$, is only ∼1.0% of the docked granules, almost significantly different from zero (*p* = 0.052, 95% C.I. (0.00003,0.035)). Again, if clustering is neglected the difference becomes (erroneously) highly significant (*p* = 0.008). Thus, we reiterate that in order to perform correct inference, clustering must be taken into account.

IRP granules have a ∼500-fold higher rate of exoytosis ($\hat{\eta }=499.5$, *p* < 10^−4^) compared to non-IRP granules, i.e., the rate-of-exocytosis during pulses was estimated to ${\hat{\rho }}_{1}=0.0012\;{\text{s}}^{-1}$ in non-IRP granules and to $\hat{\eta }{\hat{\rho }}_{1}=0.5837\;{\text{s}}^{-1}$ in IRP granules. The estimated between-pulse rate in healthy cells ${\hat{\rho }}_{2}=0.00014\;{\text{s}}^{-1}$ was ∼8 times lower than ${\hat{\rho }}_{1}$ (*p* = 0.0005). Note that these estimates compare well with the Poisson model results reported in Table 1. Interestingly, and in line with the Poisson modeling, exocytosis between pulses was estimated to occur at a higher rate in diabetic cells (${\hat{\rho }}_{2}e^{{\hat{\beta }}_{2}}=6.0\cdot 10^{-4}\;{\text{s}}^{-1}$ in diabetic cells vs. ${\hat{\rho }}_{2}=1.4\cdot 10^{-4}\;{\text{s}}^{-1}$ in healthy cells, *p* = 0.038). As typically seen for covariate effects [13], the effect of diabetes on the between-pulse hazard was estimated to be greater in the frailty formulation compared to the Poisson model without frailty.

## Discussion

The aim of this paper was to present a novel application of a well-established statistical methodology to modern cell biological data obtained with live cell imaging. To the best of our knowledge, a rigorous and statistically sound method for the analysis of exocytosis data obtained by TIRF microscopy has been lacking.

The presented method can take into account unobserved heterogeneity by the inclusion of frailties, here exemplified by a discrete frailty representing the IRP. In addition, observed covariates, here whether a cell came from a healthy or diabetic donor, can be included for example in a proportional hazards formulation. We envisage that our approach to the study of exocytosis with the use of flexible survival modeling [12] can be extended to include more complicated, time-dependent covariates [45], such as for example Ca^2+^ concentrations [46, 47] or protein levels [5, 7, 48] at the granules. Further extensions could take into consideration spatial information in addition to the temporal data [49]. The current formulation can also readily handle more complex censoring patterns than the pattern considered here, such as for example experiments interrupted at different times. Further, the method is not limited to the study of exocytosis or to endocrine cells; TIRF imaging of exocytotic events of e.g. synaptic vesicles [8] or GLUT4 vesicles in fat or muscle cells [50, 51], or of individual endocytotic events [50, 52], produce data similar to the dataset analyzed here. Moreover, the statistical methodology was here applied to data from TIRF imaging, but it is suitable for analyzing well-defined temporal cellular events recorded with any other imaging technique.

It is a well-known fact in the statistical literature [40, 42], but often not considered in biology, that ignoring clustering typically leads to underestimation of standard errors, and thus to small ‘naive’ *p*-values. Our example shows clearly this effect, which is due to the correlation between granules in the same cell: some cells are inherently ‘highly responding’, meaning that the granules in such a cell readily undergo exocytosis, while other cells are not. Ignoring this fact, would lead, for example, to rejecting the null hypotheses *π*_1_ = 0 (Table 2) or, in the Poisson formulation, *β*_0_ = 0 (Table 1). Taking into account the clustered structure of the data yields more cautious conclusions.

Our study also highlights how different statistical models can explain the data, but with different biological interpretations. The Poisson formulation assumed that all granules in a cell behave similarly, but that the rate of exocytosis is higher during the first pulse compared to the subsequent pulses. In contrast, the frailty model assumes that the rate of exocytosis for a given granule is the same in all pulses, but that the granule population is heterogeneous, since some granules belong to the IRP and have higher exocytosis rate. The latter model respects better various biological results regarding exocytosis in *β*-cells. Thus, for this kind of studies of complex cell biological questions, a close interaction between biologists and statisticians is needed in order to formulate a biologically correct model, which then serves as the basis for performing statistical inference with results that are both biologically reasonable and statistically sound.

Our application of the frailty model to human *β*-cells estimated that the IRP constitutes 2-3% of the docked granules in healthy cells, but only approximately half as many in diabetic cells. In diabetic cells, we were unable to conclude whether an IRP is present; the estimate of *π*_1_ was borderline significant (*p* = 0.052). Further studies should investigate this aspect further. Based on a cell capacitance of ∼10 pF [53], an absolute membrane capacitance of 10 fF/*μ*m^2^, and assuming a density of ∼0.8 docked granule per *μ*m^2^ membrane [54], the number of docked granules can be estimated to be ∼800/cell. Hence, we estimate that the IRP contains ∼20 granules in healthy cells, and around 10 granules in diabetic cells. The estimate in healthy cells corresponds well to the estimate of the IRP in unstimulated mouse *β*-cells [55].

The formulation of the model assumed piecewise constant baseline hazard. This formulation allowed us to perform explicit maximum-likelihood estimation, and to quantify the rate of exocytosis during and between pulses. As expected, we found that the rate of exocytosis was higher during pulses, compared to during the interval between pulses where Ca^2+^ channels are closed. This suggests that in healthy *β*-cells a close coupling between Ca^2+^ channels and insulin granules guarantees tight control of synchronized secretion. Interestingly, between pulses the rate of exocytosis was significantly higher in diabetic cells, as was the total amount of exocytosis during the experiments (Fig 2). This asynchronous release may correspond to basal insulin secretion, which is increased in diabetic mouse models [22], and even in early phases of human diabetes [56, 57], in agreement with our findings. The higher between-pulse rate might be explained by a looser coupling between Ca^2+^ channels and insulin granules in diabetic cells [22], so that residual Ca^2+^ remaining after the end of the K^+^ pulse and closure of Ca^2+^ channels triggers unsynchronized exocytosis. Such a scenario would require that the Ca^2+^ affinity for exocytosis is higher in the granules located away from the Ca^2+^ channels [58].

In summary, we have shown how to adapt time-to-event analysis to the study of TIRF imaging data of exocytosis in human *β*-cells. This powerful statistical methodology allows quantifying several biologically interesting parameters, such as rates of exocytosis, probabilities of an event in a certain time interval, and the size of the IRP, in healthy and diabetic *β*-cells. In this context, rigorous statistical tests taking into consideration the clustered structure of the data are needed to reflect the correlation between granules within the same cell. This makes it possible to correctly investigate hypotheses of disturbances in diseased cells. We believe the presented approach, which should be seen as a starting point for future extensions, could be generally applicable to analysis of a range of cell biological data with well-defined temporal events, also in the presence of more complicated covariates and censoring patterns.
